# Supplementary material for: Mercury Removal from Aqueous Solution Using ETS-4 in the Presence of Cations of Distinct Sizes
Source: Materials (Basel). 2020 Dec 22;14(1):11. doi: 10.3390/ma14010011 (PMC7792953; doi:10.3390/ma14010011)
Supplement: Supplementary file 1 [file materials-14-00011-s001.pdf]

materials

MDPI

Supplementary Materials

Mercury Removal from Aqueous Solution Using ETS-4 in the Presence of Cations of Distinct Sizes

Simão P. Cardoso [1], Tiago L. Faria [1], Eduarda Pereira [2], Inês Portugal [1], Cláudia B. Lopes [1,*] and Carlos M. Silva [1,*]

[1] CICECO, Department of Chemistry, University of Aveiro, Campus de Santiago, 3810-193 Aveiro, Portugal; simaocardoso@ua.pt (S.P.C.); tiago.luis@live.ua.pt (T.L.F.); inesport@ua.pt (I.P.)
[2] QOPNA & LAQV-REQUIMTE, Department of Chemistry, University of Aveiro, 3810-193 Aveiro, Portugal; eduper@ua.pt
* Correspondence: claudia.b.lopes@ua.pt (C.B.L.); carlos.manuel@ua.pt (C.M.S.); Tel: +351-234-401549 (C.B.L. and C.M.S.); Fax: +351-234-370084 (C.B.L. and C.M.S.)

Citation: Simão P. Cardoso; Faria, T.L.; Pereira, E.; Inês Portugal; Cláudia B. Lopes; Silva, C.M. Mercury Removal from Aqueous Solution Using ETS-4 in the Presence of Cations of Distinct Sizes. *Materials* **2021**, *14*, 11. https://doi.org/10.3390/ma14010011

Received: 18 November 2020
Accepted: 18 December 2020
Published: 22 December 2020

Publisher's Note: MDPI stays neutral with regard to jurisdictional claims in published maps and institutional affiliations.

Copyright: © 2020 by the authors. Submitted for possible open access publication under the terms and conditions of the Creative Commons Attribution (CC BY) license (http://creativecommons.org/licenses/by/4.0/).

S1. Modelling Ion Exchange Equilibrium

The ion exchange between counter ions A^{z_A} and B^{z_B} (with valences z_A and z_B) present in the solution and in the solid exchanger, respectively, can be represented as [1,2]:

$$z_B A^{z_A} + z_A \overline{B^{z_B}} \leftrightarrow z_B \overline{A^{z_A}} + z_A B^{z_B} \quad \text{(S1)}$$

where the capping bar denotes the exchanger phase, A is Hg^{2+} and B is Na^{+}. The corresponding thermodynamic equilibrium expressed in terms of ion activities is:

$$\boldsymbol{K_B^A(T)} = \frac{\bar{a}_A^{z_B} a_B^{z_A}}{a_A^{z_B} \bar{a}_B^{z_A}} \quad \text{(S2)}$$

$$\bar{a}_i = \bar{\gamma}_i \times y_i \quad \textit{and} \quad a_i = \gamma_i \times m_i \quad \text{(S3)}$$

where $K_B^A(T)$ is the equilibrium thermodynamic constant, a_i and $\bar{a}_i$ are the activities of counter ions in solution and solid exchanger, respectively, γ_i and $\bar{\gamma}$ are the activity coefficients in solution and in solid phases, respectively, y_i is the mole fraction of i in the exchanger, m_i is the molality of i in the liquid solution (mol kg^{-1}).

In the literature, several models to estimate activity coefficients have been successfully employed, such as Debye–Hückel, [3] Pitzer [3–8], and Bromley [9], for liquid solution, and Wilson [3–7,9], Margules [8], NRTL [4,8], and UNIQUAC [4,8] for the ion exchanger. In this work, the Debye– Hückel model was adopted for γ_i and the Wilson model for $\bar{\gamma}_i$. The models are summarized in Table S1 [2,5,10].

In order to optimize the equilibrium constants of the system, $K_B^A(T)$, independently of the parameters of the activity coefficients in the exchanger phase (Λ_{12} and Λ_{21}), two powerful and thermodynamically consistent approaches are available in the literature, namely Gaines and Thomas [11] and Ioannidis et al. [12]. The second method was adopted in this work, since it does not require experimental data over the entire concentration range. The Ioannidis et al. method is based on the following equation:

$$\ln\frac{\bar{\gamma}_{\mathrm{A}}^{z_{\mathrm{B}}}(\theta_2)}{\bar{\gamma}_{\mathrm{A}}^{z_{\mathrm{B}}}(\theta_1)}+\ln\frac{\bar{\gamma}_{\mathrm{B}}^{z_{\mathrm{A}}}(\theta_1)}{\bar{\gamma}_{\mathrm{B}}^{z_{\mathrm{A}}}(\theta_2)} = \ln K_{\mathrm{aB}}^{\mathrm{A}}(\theta_1)-\ln K_{\mathrm{aB}}^{\mathrm{A}}(\theta_2) \quad \text{(S4)}$$

where θ_1 and θ_2 represent two points of the equilibrium isotherm, and $K_{\mathrm{aB}}^{\mathrm{A}}$ is the corrected selectivity coefficient: $K_{\mathrm{aB}}^{\mathrm{A}} = K_{\mathrm{B}}^{\mathrm{A}}\left(\bar{\gamma}_{\mathrm{B}}^{z_{\mathrm{A}}}/\bar{\gamma}_{\mathrm{A}}^{z_{\mathrm{B}}}\right)$. One data point is fixed (*e.g.*, θ_1) while θ_2 spans the remaining ones, for which $\ln K_{\mathrm{aB}}^{\mathrm{A}}$ are calculated. Then the differences $\ln K_{\mathrm{aB}}^{\mathrm{A}}(\theta_1)-\ln K_{\mathrm{aB}}^{\mathrm{A}}(\theta_2)$ are computed, and the $\bar{\gamma}_{\mathrm{i}}$ parameters (Λ_{12} and Λ_{21}) are optimized by minimizing the sum of residuals [2].

Table S1. Models adopted for the estimation of activity coefficients of counter ions in the liquid and exchange phase.

	Model for $\boldsymbol{\gamma}_{\mathrm{i}}$		Notes
Debye-Hückel (DH)	$\mathbf{Ln}\,\boldsymbol{\gamma}_{\mathrm{i}} = -\frac{\boldsymbol{A}_{\gamma}\boldsymbol{z}_{\mathrm{i}}^{2}\sqrt{\boldsymbol{I}}}{\mathbf{1}+\boldsymbol{\beta a}\sqrt{\boldsymbol{I}}}$	(S5)	
	$\boldsymbol{A}_{\gamma} = \left(\frac{\boldsymbol{\varepsilon}^{2}}{\boldsymbol{\epsilon k}_{\mathrm{B}}\boldsymbol{T}}\right)^{3/2}\sqrt{\frac{2\pi\rho_{\mathrm{w}}N_{0}}{1000}}$ $(\mathbf{S6.1})$ **and** $\boldsymbol{\beta} = \sqrt{\frac{8\pi e^{2}N_{0}\rho_{\mathrm{w}}}{1000\epsilon k_{\mathrm{B}}T}}$ (S6.2)	(S6)	a (Å) is the minimum approximation distance between ions;<br>$\boldsymbol{n}$ is the number of charged species in solution,<br>$\boldsymbol{A}_{\gamma}$ is the Debye-Hückel constant;<br>$\boldsymbol{N}_{0} = \mathbf{6.02214086}\times\mathbf{10}^{\mathbf{23}}$ mol^{-1} is the Avogadro's constant,<br>$\boldsymbol{\rho}_{\mathbf{w}}$ (g cm^{-3}) is the density;<br>$\boldsymbol{\epsilon}$ (C^{2} N^{-1} m^{-2}) is the dielectric constant;<br>$\boldsymbol{k}_{\mathbf{B}} = \mathbf{1.38066}\times\mathbf{10}^{-\mathbf{19}}$ J K^{-1} is the Boltzmann's constant;<br>$\boldsymbol{T}$ (K) is the absolute temperature;<br>$\boldsymbol{e} = \mathbf{1.60206}\times\mathbf{10}^{-\mathbf{19}}$ C is the electron charge
	$\boldsymbol{I} = \frac{\mathbf{1}}{\mathbf{2}}\sum_{i=1}^{n}\boldsymbol{z}_{\mathrm{i}}^{2}\boldsymbol{m}_{\mathrm{i}}$	(S7)	
	Model for $\bar{\boldsymbol{\gamma}}_{\mathrm{i}}$		Notes
Wilson (WL)	$\mathbf{Ln}\,\bar{\boldsymbol{\gamma}}_{\mathbf{i}} = \mathbf{1} - \mathbf{ln}\left(\sum_{j=1}^{n} y_{\mathrm{j}}\,\boldsymbol{\Lambda}_{\mathbf{ij}}\right) - \sum_{k=1}^{n}\frac{y_{\mathrm{k}}\boldsymbol{\Lambda}_{\mathbf{ki}}}{\sum_{j=1}^{n} y_{\mathrm{j}}\boldsymbol{\Lambda}_{\mathbf{kj}}}$	(S8)	$\boldsymbol{\Lambda}_{\mathbf{ij}}$ and $\boldsymbol{\Lambda}_{\mathbf{ji}}$ are binary interaction temperature-dependent parameters defined such that $\boldsymbol{i}\neq\boldsymbol{j}$;
NRTL	$\mathrm{Ln}\,\bar{\boldsymbol{\gamma}}_{\mathrm{i}} = \frac{\sum_{j=1}^{n_{\mathrm{c}}} y_{\mathrm{j}}\boldsymbol{\tau}_{\mathrm{ji}}\boldsymbol{G}_{\mathrm{ji}}}{\sum_{k=1}^{n_{\mathrm{c}}} y_{\mathrm{k}}\boldsymbol{G}_{\mathrm{ki}}} + \sum_{j=1}^{n_{\mathrm{c}}}\frac{y_{\mathrm{j}}\boldsymbol{G}_{\mathrm{ij}}}{\sum_{k=1}^{n_{\mathrm{c}}} y_{\mathrm{k}}\boldsymbol{G}_{\mathrm{kj}}}\left(\boldsymbol{\tau}_{\mathrm{ij}} - \frac{\sum_{r=1}^{n_{\mathrm{c}}} y_{\mathrm{r}}\boldsymbol{\tau}_{\mathrm{rj}}\boldsymbol{G}_{\mathrm{rj}}}{\sum_{k=1}^{n_{\mathrm{c}}} y_{\mathrm{k}}\boldsymbol{G}_{\mathrm{kj}}}\right)$	(S9)	g_{ij} is an energy parameter characteristic of the $i-j$ interaction;<br>α_{ij} is related to the non-randomness in the mixture;<br>$\mathfrak{R} = \mathbf{8.3145}$ J mol^{-1} K^{-1} is the universal gas constant
	$\boldsymbol{\tau}_{\mathrm{ij}} = \frac{\boldsymbol{g}_{\mathrm{ij}}-\boldsymbol{g}_{\mathrm{jj}}}{\mathfrak{R}\boldsymbol{T}}$, $\boldsymbol{G}_{\mathrm{ij}} = \mathbf{exp}(-\propto_{\mathrm{ij}}\boldsymbol{\tau}_{\mathrm{ij}})$, $\propto_{\mathrm{ji}} = \propto_{\mathrm{ij}}$	(S10)	

S2. Modelling Ion Exchange Kinetics

The mass transport phenomena can be effectively described by Nernst–Planck (NP) and generalized Maxwell–Stefan (MS) equations, taking into account both concentration and electrical potential gradients. In this work, the MS formalism is adopted to model the removal performance of mercury(II) ion from aqueous solution, in view of the reliable results achieved in other research works focusing ion exchange [2,13–17]. Although frequently used, the semi-empirical pseudo-first and pseudo-second order equations for the kinetic performance of an ion exchange process possess no theoretical background, which limits their application and extrapolation [18]. However, Rodrigues and Silva [18] showed recently that, in the case of a system with a linear isotherm, the pseudo-first order equation provides equivalent results to the linear driving force model of Glueckauf.

S2.1. Modelling Ion Exchange Kinetics

Surface diffusion is the transport mechanism coherent with the small pore diameters of ETS-4 ($(3-4)\times 10^{-10}$ m). Assuming uniform distribution of fixed charges in the solid, which is taken as the $(n+1)^{\mathrm{th}}$ component and possesses null velocity ($u_{n+1} = 0$, as in the dusty gas model), the generalized MS equation of species i in multicomponent ionic system is given by [13–15,19,20]:

$$-\frac{y_i'}{\Re T}\nabla\bar{\mu}_i - y_i' z_i \frac{F}{\Re T}\nabla\bar{\varphi} = \sum_{\substack{j=1\\ j\neq i}}^{n} \frac{y_j' N_i - y_i' N_j}{q_t Đ_{ij}} + \frac{y_s' N_i}{q_t Đ_{is}} \quad \text{(S11)}$$

where $y_i' = q_i/q_t$ is the molar fraction of ion i, $y_s' = q_s/q_t$ the molar fraction of co-ions, and $q_t = q_A + q_B + q_s$ is the total molar concentration of ionic species in the exchanger (subscript *s* denotes the solid), $\nabla\bar{\mu}_i$ (J mol^{-1} m^{-1}) corresponds to the chemical potential gradient of *i* in the exchanger, $Đ_{ij}$ (m s^{-2}) is the MS counter diffusivity coefficient of *i-j* pair, $Đ_{is}$ is the MS surface diffusivity corresponding to the interaction between *i* and co-ions, N_i is the molar flux of the ionic species *i* (mol s^{-1} m^{-2}), $F = 96485.33289(59)$ C mol^{-1} is the Faraday constant, and $\nabla\bar{\varphi}$ (volt) is electric potential gradient in the solid.

The chemical potential in the exchanger ($\bar{\mu}_i$, J mol^{-1}) can be defined in terms of the hypothetical liquid solution in equilibrium, taking into account the isofugacity conditions in this case (*i.e.*, $\bar{\mu}_i = \mu_i^*$):

$$\frac{y_i'}{\Re T}\nabla\bar{\mu}_\mathrm{l} = \sum_{\substack{\mathrm{j}=1\\ \mathrm{j}\neq \mathrm{i}}}^{\mathbf{n}} \Gamma_{ij}\nabla y_j' \quad \text{with} \quad \Gamma_{ij} \equiv y_i' \frac{\partial \ln(\gamma_i^* x_i^*)}{\partial y_j'} \quad \text{(S12)}$$

where γ_i^* is the activity coefficient of counter ion *i* in the equilibrium solution, Γ_{ij} is the so-called MS thermodynamic factor, and x_i^* is the molar fraction of *i* in the hypothetical liquid solution and is related with y_i' by the equilibrium isotherm. Combining the previous equations and solving for the fluxes, N_i, a n-dimensional matrix form is obtained which introduces the well-known MS matrix, $[B]$:

$$(\boldsymbol{N}) = -\boldsymbol{q}_t[\boldsymbol{B}]^{-1}[\boldsymbol{\Gamma}](\nabla\boldsymbol{y}') - \boldsymbol{q}_t[\boldsymbol{B}]^{-1}(\nabla\bar{\boldsymbol{\xi}}) \quad \text{(S13)}$$

$$\textbf{with } B_{ii} \equiv \frac{y_s'}{Đ_{is}} + \sum_{\substack{j=1\\ j\neq i}}^{n} \frac{y_j'}{Đ_{ij}} \quad \textbf{and} \quad B_{ij} \equiv -\frac{y_i'}{Đ_{ij}} \quad \text{(S14)}$$

$$\textbf{and } \nabla\bar{\xi}_i \equiv y_i' z_i \frac{F}{\Re T}\nabla\bar{\varphi} \quad \text{(S15)}$$

The conditions of electroneutrality ($\sum_{i=1}^{n_c+1} q_i z_i = 0$) and no electric current ($\sum_{i=1}^{n_c+1} z_i N_i = 0$) are used to eliminate $\nabla\bar{\varphi}$ from the generalized MS equations, giving rise to:

$$\frac{F}{\Re T}\nabla\bar{\varphi} = -\frac{\sum_{i=1}^{n} z_i(\sum_{j=1}^{n} L_{ij}\nabla y'_j)}{\sum_{i=1}^{n} y'_i z_i(\sum_{j=1}^{n} z_j L_{ji})}, \textbf{ where } [L] = [B]^{-1} \quad \text{(S16)}$$

S2.2. Modelling Ion Exchange Kinetics

The material balance to the ion exchanger and to the whole system (liquid solution and solid) assumes: i) isothermal operation; ii) perfectly stirred tanks; iii) spherical solid particles; iv) negligible liquid and solid volume changes; v) film and intraparticle mass transfer limitations; and vi) Donnan exclusion, i.e., co-ions are excluded from the ETS-4 particles [1]. Their mathematical expressions are:

$$\left(\frac{\partial \boldsymbol{q}_{\mathrm{A}}}{\partial \boldsymbol{t}}\right) = -\frac{\mathbf{1}}{\boldsymbol{r}^2}\frac{\boldsymbol{\partial}}{\partial \boldsymbol{r}}(\boldsymbol{r}^2 \boldsymbol{N}_{\mathrm{A}}) \quad \text{(S17)}$$

$$\frac{dC_{\mathrm{A}}}{dt} = -\frac{m_{\mathrm{s}}}{\rho_{\mathrm{s}} V_{\mathrm{L}}}\frac{d\langle q_{\mathrm{A}}\rangle}{dt} \quad \text{(S18)}$$

where $q_{\mathrm{A}}\,(t,r)$ (mol m^{-3}) is the cation concentration in the exchanger (solid loading), t (s) is time, r (m) is radial position in the particle, N_{A} (mol s^{-1} m^{-2}) is internal counter ion flux, C_{A} (mol m^{-3}) is the counter ion concentration in the liquid, m_{s} (kg) is the exchanger dry-weight, ρ_{s} (kg m^{-3}) is exchanger density, and V_{L} (m^3) is the volume of liquid solution. The average loading per unit particle volume is computed by:

$$\langle q_{\mathrm{A}}(t)\rangle = \frac{3}{R_{\mathrm{p}}^3}\int_0^R r^2 q_{\mathrm{A}}\,(t,r)dr \quad \text{(S19)}$$

where R_{p} (m) is the particle radius. The initial and boundary conditions are:

$$t = 0, \quad q_{\mathrm{A}} = q_{\mathrm{A,0}} = 0 \ \text{ and } C_{\mathrm{A}} = C_{\mathrm{A,0}} \quad \text{(S20)}$$

$$r = R_{\mathrm{p}}, \quad q_{\mathrm{A}} = q_{\mathrm{A,R}} \quad \text{(S21)}$$

$$r = 0, \quad \left(\frac{\partial q_{\mathrm{A}}}{\partial r}\right) = 0 \quad \text{(S22)}$$

with $q_{\mathrm{A,R}}$ (mol m^{-3}) being the ion concentration at particle surface, which is calculated by equalizing the internal diffusion and the external convective fluxes at surface ($r = R_{\mathrm{p}}$):

$$N_{\mathrm{A}}|_{r=R} = k_{\mathrm{f}}\,(C_{\mathrm{A}} - C_{\mathrm{A,R}}) \quad \text{(S23)}$$

The convective mass transfer coefficient, k_{f} (m s^{-1}), and the fluid concentration at particle surface $C_{\mathrm{A,R}}$ (mol m^{-3}) may be related to $q_{\mathrm{A,R}}$ by an isotherm, since they are in equilibrium.

2.3. Semi-Empirical Equations

The kinetic data were also fitted using two of the most popular expressions in environmental science, namely the pseudo-first and pseudo-second order equations [21,22]:

$$\langle q_{\mathrm{A}}\rangle = q_{\mathrm{A,e}}(1 - e^{-k_1 t}) \quad \text{(S24)}$$

$$\langle q_{\mathrm{A}}\rangle = q_{\mathrm{A,e}}^2\frac{k_2 t}{1 + q_{\mathrm{A,e}} k_2 t} \quad \text{(S25)}$$

where $q_{\mathrm{A,e}}$ is the concentration of Hg^{2+} in the exchanger phase in equilibrium with the fluid phase with concentration $C_{\mathrm{A,e}}$, and k_1 (h^{-1}) and k_2 (m^3 mol^{-1} h^{-1}) are the sorption rate constants of each model.

References

1. Helfferich, F.G. *Ion Exchange*; Dover Publications: New York, NY, USA, 1962.
2. Lito, P.F.; Cardoso, S.P.; Loureiro, J.M.; Silva, C.M. Ion Exchange Equilibria and Kinetics. In *Ion Exchange Technology I—Theory and Materials;* Inamuddin, D., Luqman, M., Eds.; Springer: Dordrecht, Netherlands, 2012; pp 51–120.

Cardoso, S.P.; Azenha, I.S.; Lin, Z.; Portugal, I.; Rodrigues, A.E.; Silva, C.M. Experimental measurement and modeling of ion exchange equilibrium and kinetics of cadmium(II) solutions over microporous stannosilicate AV-6. *Chem. Eng. J.* **2016**, *295*, 139–151.

Aniceto, J.P.S.; Lito, P.F.; Silva, C.M. Modeling Sorbent Phase Nonideality for the Accurate Prediction of Multicomponent Ion Exchange Equilibrium with the Homogeneous Mass Action Law. *J. Chem. Eng. Data* **2012**, *57*, 1766–1778.

Vo, B.S.; Shallcross, D.C. Modeling Solution Phase Behavior in Multicomponent Ion Exchange Equilibria Involving H^+, Na^+, K^+, Mg^{2+}, and Ca^{2+} Ions. *J. Chem. Eng. Data* **2005**, *50*, 1995–2002.

Mehablia, M.A.; Shallcross, D.C.; Stevens, G.W. Ternary and Quaternary Ion Exchange Equilibria. *Solvent Extr. Ion Exch.* **1996**, *14*, 309–322.

Valverde, J.L.; De Lucas, A.; GonzáLez, M.; Rodríguez, J.F. Ion-Exchange Equilibria of Cu^{2+}, Cd^{2+}, Zn^{2+}, and Na^+ Ions on the Cationic Exchanger Amberlite IR-120. *J. Chem. Eng. Data* **2001**, *46*, 1404–1409.

Aniceto, J.P.S.; Cardoso, S. P.; Faria, T.L.; Lito, P.F.; Silva, C.M. Modeling ion exchange equilibrium: Analysis of exchanger phase non-ideality. *Desalination* **2012**, *290*, 43–53.

Borba, C.E.; Silva, E.A.; Spohr, S.; Santos, G.H.F.; Guirardello, R. Ion Exchange Equilibrium Prediction for the System Cu^{2+}-Zn^{2+}-Na^+. *J. Chem. Eng. Data* **2010**, *53*, 1333–1341.

3. Zemaitis, J.F.; Clark, D.M.; Rafal, M.; Scricvner, N.C. *Handbook of Aqueous Electrolyte Thermodynamics: Theory & Application*; Wiley: New York, NY. USA, 1986.

Gaines, G.L.; Thomas, H.C. Adsorption Studies on Clay Minerals. II. A Formulation of the Thermodynamics of Exchange Adsorption. *J. Chem. Phys.* **1953**, *21*, 714–718.

Ioannidis, S.; Anderko, A.; Sanders, S.J. Internally consistent representation of binary ion exchange equilibria. *Chem. Eng. Sci.* **2000**, *55*, 2687–2698.

Lito, P.F.; Aniceto, J.P.S.; Silva, C.M. Maxwell–Stefan based modelling of ion exchange systems containing common species (Cd^{2+}, Na^+) and distinct sorbents (ETS-4, ETS-10). *Int. J. Environ. Sci. Technol.* **2013**, *12*, 183–192.

Silva, C.M.; Lito, P. F. Application of the Maxwell–Stefan approach to ion exchange in microporous materials. Batch process modelling. *Chem. Eng. Sci.* **2007**, *62*, 6939–6946.

Lito, P.F.; Aniceto, J.P.S.; Silva, C.M. Modelling ion exchange kinetics in zeolyte-type materials using Maxwell-Stefan approach. *Desalin. Water Treat.* **2013**, *52*, 5333–5342.

Lito, P.F.; Silva, C.M. Comparison between Maxwell-Stefan and Nernst-Planck Equations to Describe Ion Exchange in Microporous Materials. *Defect Diffus. Forum* **2008**, *273–276*, 776–781.

Sun, Y., Yang, K. Analysis of mass transport models based on Maxwell–Stefan theory and Fick's law for protein uptake to porous anion exchanger. *Sep. Purif. Technol.* **2008**, *60*, 180–189.

Rodrigues, A.E.; Silva, C.M. What's wrong with Lagergreen pseudo first order model for adsorption kinetics? *Chem. Eng. J.* **2016**, *306*, 1138–1142.

Krishna, R.; Wesselingh, J. A. The Maxwell-Stefan approach to mass transfer. *Chem. Eng. Sci.* **1997**, *52*, 861–911.

Lito, P.F.; Zhou, C.F.; Santiago, A.S.; Rodrigues, A.E.; Rocha, J.; Lin, Z.; Silva, C.M. Modelling gas permeation through new microporous titanosilicate AM-3 membranes. *Chem. Eng. J.* **2010**, *165*, 395–404.

Lagergren, S.Y. About the theory of so-called adsorption of soluble substances. *K. Sven. Vetenskapsakademiens. Handl.* **1898**, *24*, 1–39.

Ho, Y.; McKay, G. Pseudo-second order model for sorption processes. *Process Biochem.* **1999**, *34*, 451–465.
